# Allo-Specific Humoral Responses: New Methods for Screening Donor-Specific Antibody and Characterization of HLA-Specific Memory B Cells

**DOI:** 10.3389/fimmu.2021.705140

**Published:** 2021-07-13

**Authors:** Shengli Song, Miriam Manook, Jean Kwun, Annette M. Jackson, Stuart J. Knechtle, Garnett Kelsoe

**Affiliations:** ^1^ Department of Immunology, Duke University School of Medicine, Durham, NC, United States; ^2^ Department of Surgery, Duke University School of Medicine, Durham, NC, United States

**Keywords:** donor specific antibody (DSA), B cell, sensitization, transplantation, antibody mediated rejection (AMR)

## Abstract

Antibody-mediated allograft rejection (AMR) causes more kidney transplant failure than any other single cause. AMR is mediated by antibodies recognizing antigens expressed by the graft, and antibodies generated against major histocompatibility complex (MHC) mismatches are especially problematic. Most research directed towards the management of clinical AMR has focused on identifying and characterizing circulating donor-specific HLA antibody (DSA) and optimizing therapies that reduce B-cell activation and/or block antibody secretion by inhibiting plasmacyte survival. Here we describe a novel set of reagents and techniques to allow more specific measurements of MHC sensitization across different animal transplant models. Additionally, we have used these approaches to isolate and clone individual HLA-specific B cells from patients sensitized by pregnancy or transplantation. We have identified and characterized the phenotypes of individual HLA-specific B cells, determined the V(D)J rearrangements of their paired H and L chains, and generated recombinant antibodies to determine affinity and specificity. Knowledge of the BCR genes of individual HLA-specific B cells will allow identification of clonally related B cells by high-throughput sequence analysis of peripheral blood mononuclear cells and permit us to re-construct the origins of HLA-specific B cells and follow their somatic evolution by mutation and selection.

## Introduction

HLA sensitization remains a barrier to transplantation of all organ types (1, 2). The etiology of allo-antibodies stem from exposure to foreign HLA through pregnancy, transfusion, and previous transplantation and is driven by the high polymorphism among HLA genes. Advances in histocompatibility testing have improved the sensitivity and specificity of HLA antibody detection and has revealed broader HLA sensitization among transplant candidates. Transplantation in the presence of donor-specific HLA antibody (DSA) for mismatched HLA determinants in the donor’s phenotype increases the risk of rejection and allograft loss, while locating an HLA compatible donor can increase waiting time and associated mortality (3).

Screening for HLA antibodies is routinely performed prior to transplantation to avoid DSA and the increased risk for AMR and graft failure. In this way, donor selection and transplant outcomes are optimized. HLA single antigen bead (SAB) Luminex® assays allow sensitive screening for antibodies specific for hundreds of HLA alleles and are used widely in clinical practice. These assays, however, are prohibitively expensive for non-clinical studies and unavailable for animal species frequently used to study humoral responses to MHC antigens, notably macaques and swine. This lack of comparable diagnostic tools limits detailed study of the breadth and specificity of MHC-specific humoral responses in non-human transplant model systems. Crossmatch assays using donor lymphocytes have been used to detect the presence or absence of species-specific anti-MHC antibodies in experimental transplantation studies with rhesus macaques (4) and swine (5). In a series of recent studies (6, 7), swine SLA class II single antigen expressing HEK293 cell lines were generated to monitor xeno-reactive antibody responses with human serum samples, albeit, with singleplex capability and limited MHC allele coverage.

Sensitized patients are vulnerable to AMR when transplanted in the presence of circulating DSA, but some may also experience early AMR as a result of post-transplant activation of cryptic HLA-specific B memory. Unfortunately, current methods of assessing AMR risk at time of transplant relies solely on detection of circulating DSA and are unable to detect HLA-primed memory B (Bmem) cells that can rapidly proliferate and differentiate to antibody secreting cells on restimulation. In order to reveal this hidden humoral memory, a number of groups have developed *in vitro* methods to evaluate the presence of HLA-specific B cells capable of alloantibody production on stimulation. Luque et al. have demonstrated “Flourospot” techniques that permit semi-quantitative, multiplex detection of HLA specific B cells that secrete antibody following B cell stimulation (8). Karahan et al. demonstrated a bulk-culture method stimulating PBMC with TLR8/9 ligands and IL-2 to elicit HLA antibody production and screening culture supernatant using a commercially available SAB Luminex® platform (9). These methods demonstrate the ability to detect HLA-specific humoral memory, likely residing in the Bmem cell compartments; yet the exact *in vivo* frequency and phenotype of DSA B cells, and the characteristics of DSA B-cell antigen receptors (BCRs) remain obscure. A recent study by Heidt and colleagues has moved the field a step forward by using HLA tetramers to sort single HLA-specific B cells to allow more accurate characterization of their frequency and BCR structural analysis (10).

Here we present new tools developed to aid in the identification and characterization of B cells sensitized by allo-MHC exposures and to advance research in human and animal transplantation models. In our previous studies with influenza-vaccinated subjects, a single B-cell culture method was established to profile hemagglutinin specific Bmem from PBMC samples (11). This culture system supports the proliferation and differentiation of single naïve and Bmem cells and secretion of clonal antibody into culture supernatants. The amount of antibody secreted is sufficient so that antibody specificities are readily determined with the culture supernatants, while the V(D)J DNA rearrangements encoding BCRs can be recovered from expanded B-cell clones in culture wells for sequencing and re-expression. For antibody screening of the culture supernatants, we developed a cell-based immunoassay platform for the detection of antibody binding to cell-surface expressed antigens in a multiplex way (submitted). In this study, we further adapted this cellular platform and established an economic, versatile, and multiplex reporter cell assay for the detection of antibodies specific for MHC molecules from different mammalian species. Coupled with the established single Bmem cell culture system, we identified and characterized an anti-HLA B memory clone from one allo-sensitized patient.

## Materials and Methods

### Mice, Rhesus Macaques and Human Subjects

Female C57BL/6 mice were obtained from the Jackson Laboratory and maintained under specific-pathogen-free conditions at the Duke University Animal Care Facility. Splenocytes were isolated from one 12-week-old mouse for RNA extraction. All experiments involving animals were approved by the Duke University Institutional Animal Care and Use Committee (IACUC A128-20-06).

Serum samples from rhesus macaques were obtained from animals sensitized by serial skin transplantation as part of an established transplant model (12). Naïve juvenile male rhesus macaques received two swapping skin transplants from their maximally Mamu mismatched donor, at eight-week intervals. Day 0 samples refer to naïve sera, while "Day 54-70 represent ‘peak’ samples, taken 2 weeks after the second skin transplant (IACUC A153-18-06).

Peripheral blood mononuclear cells (PBMCs) were obtained from one human subject under Duke Institutional Review Board Committee guidelines (IRB Pro00062495) and archived sera from transplant recipients obtained under IRB Pro00104220.

### HLA Typing and HLA Antibody Analyses

High‐resolution HLA typing was performed by next generation sequencing using the MIA FORA kit (Immucor, Inc., Norcross, GA, USA) on an Illumina Miseq platform (Illumina, Inc., San Diego, CA, USA). HLA specific antibodies were evaluated using HLA single antigen Luminex^®^ beads (SAB) according to manufacturer’s instructions and acquired on a LABScreen 200 instrument (One Lambda, Canoga Park, CA). HLA antibody positivity was determined by HLA pattern analysis incorporating serological cross-reactive patterns and HLA matchmaker eplet analysis FUSION version 4.3 (One Lambda, Canoga Park, CA) and HLA Epitope Registry (https://www.epregistry.com.br/). Recombinant antibodies: P3E7 and P3E7-UA were tested at 0.16 mg/ml and P3E7 was also tested at 0.96 mg/ml (~6x) and analyzed as described above. Three-dimensional structures of the HLA-A molecules were created using Cn3d software (https://www.ncbi.nlm.nih.gov/Structure/CN3D/cn3d.shtml).

### Original Plasmids and DNA Templates for Gene Cloning

pU6-(BbsI)_CBh-Cas9-T2A-BFP (Addgene plasmid # 64323) was a gift from Ralf Kuehn (13). pLV-EF1a-IRES-Puro (Addgene plasmid # 85132) was a gift from Tobias Meyer (14). pMD2.G (Addgene plasmid # 12259) and psPAX2 (Addgene plasmid # 12260) were gifts from Didier Trono. Human IgG1 expression vector AbVec2.0-IGHG1 (Addgene plasmid # 80795), human IgK expression vector AbVec1.1-IGKC (Addgene plasmid # 80796) and human IgL expression vector AbVec2.1-IGLC2-MscI (Addgene plasmid # 80797) were gifts from Hedda Wardemann (15, 16). The lentiviral transfer vector plasmids pLB-EF1a, pLB-EXIP, pLB-EF1a-IRES-mCD86, pLB-EF1a-EBFP2, pLB-EF1a-mTurquoise2, pLB-EFS-mNeonGreen and pLB-EF1a-mCardinal were generated previously (submitted). Plasmid mNeonGreen-C1 was provided by Allele Biotechnology and Pharmaceuticals Inc. under a license for non-commercial use.

DNA templates coding 14 HLA alleles were synthesized (Gene Universal Inc.) based on amino acid sequences in the IPD-IMGT/HLA database (https://www.ebi.ac.uk/ipd/imgt/hla/), including HLA-A*01:01, A*02:01, A*03:01, A*24:02, B*07:02, B*15:01, B*27:05, B*35:01, B*44:02, B*57:01, DRA*01:01/DRB1*04:01, DQA1*05:01/DQB1*02:01, DQA1*04:01/DQB1*04:02 and DQA1*05:03/DQB1*03:01. For class II molecules, the coding sequences for α and β chains were linked with coding sequences for a T2A peptide (17), which can induce ribosomal skipping during translation. DNA templates coding following molecules were also synthesized (Gene Universal Inc.) based on amino acid sequences in GenBank database, including mouse IAb (H2-Aa (GenBank Accession NM_010378.3) and H2-Ab1 (NM_207105.3) linked with a T2A peptide), mouse CD74 (NM_010545), human CD74 (NM_001025159.2) and rhesus β2M (NP_001040602.1). Plasmids containing coding sequences for eight class I Mamu alleles were provided by Dave O’Connor (University of Wisconsin-Madison), including Mamu-A1*001:01, A1*002:01, A1*004:01, A1*006:02, A1*023:01, B*001:01, B*012:01 and B*017:01. Coding sequences for mouse H2Kb, β2M and cytoplasmic domain truncated CD86 (amino acid 1-268) were cloned from splenocyte cDNA samples from one C57BL/6 mouse. Coding sequences for human β2M were cloned from PBMC cDNA samples from one healthy donor. Rhesus IgG1 and IgK constant region sequences were cloned from PBMC cDNA samples from a rhesus monkey.

### Plasmid Modification and Gene Cloning

Standard molecular cloning procedures were followed for plasmid modification and gene cloning. Endotoxin-free plasmids were prepared (E.Z.N.A.^®^ Endo-free Plasmid DNA Mini Kit II, Omega Bio-tek) for mammalian cell transfection. All plasmids were verified by DNA Sanger sequencing (Duke University DNA Analysis Facility).

CRISPR-Cas9 targeting plasmid pCRISPRpuro was generated by replacing EBFP2 coding sequences in plasmid pU6-(BbsI)_CBh-Cas9-T2A-BFP with puromycin-resistance gene sequences from plasmid pLV-EF1a-IRES-Puro. The empty lentiviral transfer vector plasmids pLB-EXIG and pLB-EXIN were generated by replacing the puromycin-resistance gene sequences in plasmid pLB-EXIP with mNeonGreen coding sequences from plasmid mNeonGreen-C1, or neomycin-resistance gene sequences from plasmid pIRESneo2 (Clontech), respectively. Rhesus IgG1 expression vector pAbVec2.1-RhIgG1 and IgK expression vector pAbVec2.1-RhIgK were generated by replace the human IgL constant region sequences in AbVec2.1-IGLC2-MscI with rhesus IgG1 and IgK constant region sequences, respectively.

Coding sequences for H2Kb alone or H2Kb and mouse β2M linked with a T2A peptide (H2Kb-T2A-mB2M) were cloned into plasmid pLB-EXIG. H2Kb dtSCT cassettes (18) presenting OVA257 peptide (SIINFEKL) and VSV8 peptide (RGYVYQGL) were cloned into plasmid pLB-EXIP. Mouse IAb coding sequences (H2-Aa and H2-Ab1 linked with a T2A peptide) were cloned into plasmid pLB-EF1a. Coding sequences for human CD74, mouse CD74 and a chimeric mouse CD74 (ΔmCD74.Eα52, with the core CLIP peptide KPVSQMRMATPLLMRPM replaced with Eα52 peptide ASFEAQGALANIAVDKA) were cloned into plasmid pLB-EF1a-IRES-mCD86. Coding sequences for class I and class II HLA alleles and class I Mamu alleles were cloned into plasmid pLB-EXIP. Coding sequences for human and rhesus β2M molecules were cloned into plasmid pLB-EXIN.

### Culture, Transfection and Transduction of Mammalian Cell Lines

The cell lines used included Raji (ATCC CCL-86), HEK 293T (ATCC CRL-11268), and hybridoma cell lines GAP A3 (ATCC HB-122), W6/32 (ATCC HB-95) and BB7.2 (ATCC HB-82). K530 cell line was generated previously (submitted) from K562 cell line (ATCC CCL-243) by knocking out human *CD32A* gene with standard CRISPR-Cas9 technology. HEK 293T and GAP A3, W6/32 and BB7.2 hybridoma cells were cultured in DMEM medium (Gibco) supplemented with 10% heat-inactivated HyClone FBS (Cytiva), 10 mM HEPES buffer and 55 µM 2-Mercaptoethanol (all Gibco). Raji, K530 and derivative cell lines were maintained in RPMI-1640 medium (Gibco) supplemented with 10% heat-inactivated HyClone FBS, 10 mM HEPES buffer, 1 mM sodium pyruvate, 1× MEM NEAA, 55 µM 2-mercaptoethanol, 100 units/ml penicillin and 100 µg/ml streptomycin (all Gibco). For all K530 derivative cell lines, unless otherwise indicated, monoclonal cell lines were established by single-cell sorting (see below) and used in binding assays in this study.

Single-guide RNAs (sgRNAs) targeting human *B2M* exon 1 were designed with the online tool (http://crispr.mit.edu). sgRNAs used in this study were sgRNA-hB2M-1 (GGGCCGAGATGTCTCGCTCCG), sgRNA-hB2M-2 (GGAGTAGCGCGAGCACAGCTA) and a negative control sgRNA (TGTCATGCGTCACTTAGTGC). Corresponding DNA oligos were synthesized and cloned into plasmid pCRISPRpuro. K530 cells were transfected with CRISPR-Cas9 targeting plasmids using Lipofectamine 3000 Transfection Reagent (Invitrogen). Twenty-four hours after transfection, puromycin was added (2 µg/ml) for a pulse-selection. Two days later, cells were transferred to puromycin-free medium and cultured for another seven days before flow cytometry analysis and single-cell sorting (see below).

Lentiviral transfer vector plasmids were co-transfected into HEK 293T cells with packaging plasmids pMD2.G and psPAX2 using Lipofectamine 3000 Transfection Reagent (Invitrogen). Forty-eight hours after transfection, culture supernatants were harvested and filtered through 0.45-µm PVDF membrane filters (Millipore). K530 derivative cell lines were transduced with the filtered supernatants containing lentiviral vectors by spinoculation at 1000× *g* for 45 min at 32°C. For transductions with pLB-EXIP-based vectors, cells were selected with puromycin (Sigma, 2 µg/ml) between 3-7 days after transduction. Seven days after transduction, the cells were harvested for flow cytometry analysis and single-cell sorting (see below).

### Antibodies and Staining Reagents

Monoclonal antibodies used in this study included: APC-conjugated anti-human β2M (hβ2M-APC, clone 2M2, BioLegend 316312), HLA-ABC-PE-Cy7 (clone W6/32, BioLegend 311430), HLA-DR/DP/DQ-PE-Cy7 (clone Tü39, BioLegend 361708), hCD74-PE (clone LN2, BioLegend 326808), hβ2M-PE (clone 2M2, BioLegend 316306), H2Kb-AF647 (clone AF6-88.5, BioLegend 116512), H2Kb/H2Db-PE (clone 5041.16.1, ThermoFisher MA5-18000), SIIN-H2Kb-PE (clone 25-D1.16, BioLegend 141604), mCD86-PE (mouse CD86, clone GL1, BioLegend 105007), IAb-PE (clone AF6-120.1, BioLegend 116407), mCD74-AF647 (clone In1/CD74, BioLegend 151003), CLIP-PE (clone cerCLIP.1, Santa Cruz sc-12725-PE), Ea52/IAb-PE (clone YAe, Santa Cruz sc-32247-PE), Anti-mCD86-PE-Vio770 (clone PO3.3, Miltenyi Biotec 130-105-135), mIgG2a-PE (clone RMG2a-62, BioLegend 407108), HLA-DQ-PE (clone HLADQ1, BioLegend 318105), HLA-ABC (clone W6/32, BioLegend 311402), HLA-A2 (clone BB7.2, BioLegend 343302), HLA-A2 (One Lambda 0791HA), HLA-B7 (clone BB7.1, BioLegend 372402), HLA-B27 (clone HLA-ABC-m3, Sigma MAB1285), HLA-DQ/DP/DR (clone Tü39, BioLegend 361702), HLA-DR (clone L243, BioLegend 307602), HLA-DQ (clone Tü169, BioLegend 361502), hCD19-PE-Cy7 (clone HIB19, BioLegend 302216), hCD3-PE-Cy5 (clone UCHT1, BD 555334), hCD14-TriColor (clone TuK4, Invitrogen MHCD1406), hCD16-PE-Cy5 (clone 3G8, BD 555408), hCD24-BV510 (clone ML5, BioLegend 311126), hCD27-BV421 (clone M-T271, BioLegend 356418), hIgM-FITC (clone MHM-88, BioLegend 314506), hIgD-APC-Cy7 (clone IA6-2, BioLegend 348218), hIgG-APC (clone G18-145, BD 550931). Antibody GAP A3 was purified (11) from hybridoma cell line culture supernatants after adaptation into suspension culture in Expi293 Expression Medium (Invitrogen).

Isotype control antibodies included: Mouse IgG1 kappa-PE (clone MOPC-21, BioLegend 400112), Mouse IgG1 kappa-APC (clone MOPC-21, BioLegend 400122), Mouse IgG2a kappa-PE (clone MOPC-173, BioLegend 400212), Mouse IgG2a kappa-PE-Cy7 (clone MOPC-173, BioLegend 400254), Rat IgG2a kappa-PE (clone RTK2758, BioLegend 400508), Rat IgG2b kappa-AF647 (clone RTK4530, BioLegend 400626), Human IgG1 kappa (hIgG1K, Southern Biotech 0151K-01), Human IgG1 lambda (hIgG1L, Southern Biotech 0151L-01), Rhesus Monkey IgG (RhIgG, Southern Biotech 0135-01). Secondary antibodies included: Goat Anti-Human IgG-PE (Southern Biotech 2040-09), Goat Anti-Mouse IgG, Human ads-PE (Southern Biotech 1030-09), Mouse Anti-Monkey IgG-PE (clone SB108a, Southern Biotech 4700-09). Other staining reagents included: 7-AAD (BD 559925), Tetramer-PE (T-Select HLA-A*02:01 HIV gag Tetramer-SLYNTVATL-PE, MBL TB-M027-1).

### Cell Surface and Intracellular Staining, Flow Cytometry Analysis and Single-Cell Sorting and Cloning

For cell surface staining, cultures of K530 and derivative cells were harvested, centrifuged at 300× *g* for 2 min at 4°C and resuspended in staining buffer (PBS supplemented with 2% heat-inactivated FBS). After incubation with antibodies at 4°C in the dark for 30 min, cells were washed with staining buffer and resuspended in staining buffer for either secondary staining following the same procedure above or stored on ice for flow cytometry analysis or single-cell sorting. For intracellular staining, Fixation/Permeabilization Solution Kit (BD 554714) was used and manufacturer’s instructions were followed.

Flow cytometry analysis was carried out using BD FACSCanto II cytometer (Duke Cancer Institute Flow Cytometry Shared Resource). Single-cell sorting was performed with BD Aria II (The Duke Human Vaccine Institute Research Flow Cytometry Facility). The bulk cell line after transfection or transduction were labeled with corresponding antibodies and single cells expressing FP or antigen of interest were sorted into 96-well flat-bottom plates containing 100 µl/well of the complete RPMI medium above supplemented with 20% heat-inactivated FBS. Nine days after sorting, robustly proliferating cell clones were transferred into 24-well plates for further expansion. Three days later, individual monoclonal cell lines were validated for the expression of FP or antigen of interest and a single clone with a uniform expression level was selected for further engineering or used as a reporter cell line in immunoassays.

### Multiplex Reporter Cell Assay

Fluorescence-barcoded reporter cell lines expressing unique MHC antigens were pooled and labeled with reference antibodies, diluted serum samples or single B-cell culture supernatants. After a secondary labeling with PE-conjugated secondary antibody, the binding activities were detected by flow cytometry as described above. To increase the throughput, 96-well V-bottom plates were used for staining and a high-throughput sampler was used during flow cytometer detection.

### Single Bmem Cell Sorting and Culture

Human Bmem cells were isolated by flow cytometry and single B-cell cultures were established as described (11). Briefly, frozen-thawed PBMCs were stained with antibody cocktails containing hCD3-PE-Cy5, hCD14-TriColor, hCD16-PE-Cy5, hCD19-PE-Cy7, hCD24-BV510, hCD27-BV421, hIgM-FITC, hIgD-APC-Cy7, hIgG-APC, 7-AAD and HLA-A2 tetramer-PE. Antigen-specific Bmem cells were identified as 7-AAD^–^ CD3^–^ CD14^–^ CD16^–^ CD19^+^ CD24^hi^ CD27^+^ IgM^–^ IgD^–^ IgG^+^ Tetramer^+^. Single antigen-specific B-cells were sorted into 96-well culture plates with MS40L^low^ feeder cells (19, 20) and in the presence cytokines, including human IL-2, IL-4, IL-21 and BAFF (all Peprotech). After 25 days’ culture, supernatants were harvested for screening the reactivity of clonal IgG Abs. Expanded clonal B cells were frozen for V(D)J sequence analyses. IgG^+^ culture supernatants were tested for HLA binding activities with HLA-expressing cell lines in a multiplex reporter cell assay.

### BCR Rearrangement Amplification and Analysis

Rearranged V(D)J gene sequences for human Bmem cells from single-cell cultures were obtained as described (11). V(D)J rearrangements were identified with IMGT/V-QUEST (http://www.imgt.org/IMGT_vquest/) (21). Unmutated ancestor (UA) sequences were inferred with Cloanalyst (22).

### Recombinant IgG Expression and Purification

Rhesus IgG1 and human IgG1 versions of W6/32 and human IgG1 version of BB7.2 antibody expression vectors were prepared by cloning the heavy and light chain V(D)J sequences (16) from hybridoma cell line cDNA samples to corresponding expression vectors mentioned above. V(D)J sequences of HLA-specific Bmem cell clone H02P3E7 and the inferred UA (synthesized, Gene Universal Inc.) were cloned into human IgG1 and IgK expression vectors as described (15). Recombinant antibodies were produced and purified as described (11).

### Luminex® Assay

Luminex® beads conjugated with anti-human IgG were incubated with serial dilutions of IgG^+^ culture supernatants or recombinant human IgG1 antibodies. After washing, either PE-conjugated goat anti-human IgG or PE-conjugated HLA-A2 tetramers were added as the detection reagents. The concentrations of bead-bound human IgG1 antibodies were normalized to a commercial isotype control antibody and the antigen binding activities of the bound antibodies were revealed by corresponding MFI values in the tetramer-PE staining group.

## Results

### Generation of a Versatile Cell Line for Surface Expression of MHC Class I and II Molecules From Different Mammalian Species

We recently developed (submitted) the K530 cell line, a CD32A-deficient derivative of K562 cells, which was subsequently used as the parental cell line for endogenous barcoding by fluorescent proteins (FPs) and cell surface antigen expression. The parental K562 line was derived from a chronic myelogenous leukemia and does not express HLA class I and class II products (23) but is positive for β2M.

Concerned that this endogenous human β2M could interact with transduced MHC class I α chain from non-human species and generate chimeric class I molecules with altered structure and epitopes (24–26), we further modified the K530 cell line by knocking out the human *B2M* gene using a standard CRISPR-Cas9 mediated gene targeting strategy. Briefly, K530 cells were transfected with a CRISPR-Cas9 targeting vector co-expressing Cas9 nuclease and single-guide RNAs towards exon 1 region of human *B2M* gene (**Figure S1A**). After puromycin selection, more than 70% of surviving cells showed decreased intracellular β2M expression comparable to that of K530 cells stained with an isotype control antibody (**Figure S1B**). A monoclonal cell line, K528, was generated by single-cell flow cytometry sorting. The lack of β2M expression in K528 cells was confirmed by intracellular staining (**Figure S1B**), and the inactivation of *B2M* genes validated by genomic DNA sequencing (**Figure S1C**).

**Figure 1:**
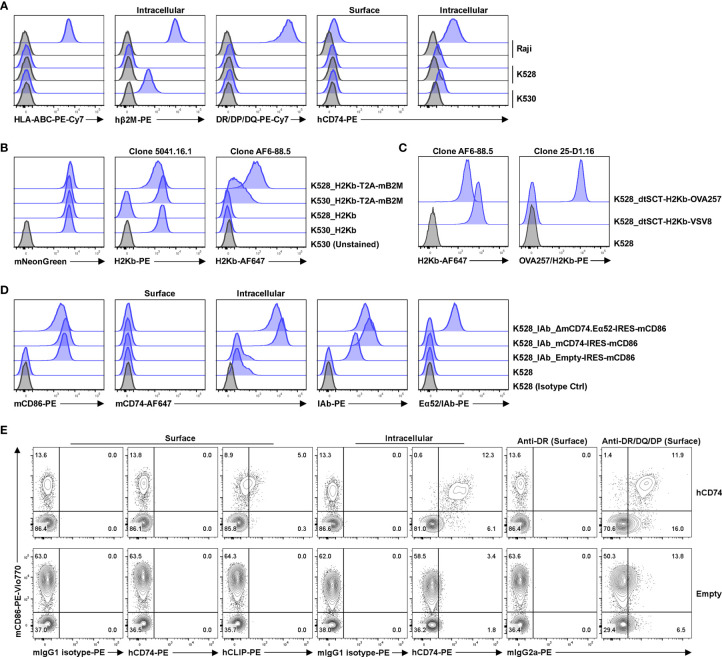
K528 is a versatile cell line for transgenic expression of MHC class I and II molecules from different mammalian species. **(A)** Phenotypic analysis of K528 cell line. K530 and Raji cells were used as controls in straining. The gating strategy is shown in **Figure S2**; similar gating strategies were applied to other panels below. Grey histograms show staining with isotype control antibodies. **(B)** K528 and K530 cells were transduced to express H2Kb with or without co-expressed mouse β2M molecules. The expression vectors co-express mNeonGreen as a selection marker. Monoclonal cell lines with comparable mNeonGreen fluorescence intensities were selected for comparison. Cell surface expression of H2Kb molecules were detected with either mouse β2M-independent (clone 5041.16.1) or -dependent (clone AF6-88.5) antibodies. **(C)** K528 cells were transduced to express single-chain trimer with disulfide trap (dtSCT) structures of H2Kb molecules with either VSV8 or OVA257 peptides. Surface expression of H2Kb and H2Kb molecules presenting OVA257 peptides were detected with relevant antibodies. **(D)** K528 cells were transduced to express IAb molecules. The resultant monoclonal cell line was further transduced with lentiviral vectors with expression cassettes Empty-IRES-mCD86 (K528-IAb), mCD74-IRES-mCD86 (K528-IAb-mCD74) or ΔmCD74.Eα52-IRES-mCD86 (K528-IAb-ΔmCD74.Eα52) in which the core CLIP sequences were replaced with IAb presenting peptide Eα52 from IEd molecule. Monoclonal cell lines expressing comparable levels of mCD86 were selected for analysis. Surface and intracellular mCD74, surface IAb and Eα52/IAb complex were detected by FACS. **(E)** K528 cells expressing HLA-DQA1*05:01/DQB1*02:01 were transduced with lentiviral vectors with expression cassettes hCD74-IRES-mCD86 (hCD74) or Empty-IRES-mCD86 (Empty). Bulk cell lines after transduction were used for detection by FACS.

The resultant clonal cell line, K528, is negative for HLA class I, class II and β2M but expresses low levels of cytoplasmic CD74 (**Figure 1A**, **Figure S2**). The K528 line can readily be made to express MHC molecules from any mammalian species. To demonstrate this versatility, the mouse class I molecule H2Kb was transduced in either K530 or K528 cells, *i.e*., in the presence or absence of human β2M, respectively. The β2M-independent H2Kb mAb (clone 5041.16.1), recognizes transduced H2Kb comparably on K530 (human β2M+) cells transduced with H2Kb alone or with co-expressed mouse β2M; on transduced K528 (human β2M–) cells, the 5041.16.1 mAb binds only to cells expressing both H2Kb and mouse β2M (**Figure 1B**). In contrast, the mouse β2M-dependent H2Kb mAb (clone AF6-88.5), recognizes only cells co-transduced with H2Kb and mouse β2M. Significantly, AF6-88.5 mAb binding to co-transduced K530 was significantly reduced by the presence of human β2M, suggesting that the endogenous human β2M efficiently competes with transgenic mouse β2M to generate chimeric class I molecules (compare 5041.16.1 binding to AF6-88.5 binding). Only K528 cells co-expressing mouse β2M supported normal expression of surface H2Kb molecules as determined by the mouse β2M-dependent H2Kb-specific antibody (**Figure 1B**). In the absence of endogenous human β2M, pMHC molecules from other species with fixed peptide can also be expressed by K528 cells as single-chain trimers with disulfide traps (dtSCT) (18) (**Figure 1C**). For a mouse class II molecule IAb, co-expression of mouse CD74 increased the surface expression level dramatically. A chimeric mouse CD74 molecule with the core CLIP peptide replaced with an exogenous antigenic peptide drove the presentation of corresponding peptide by IAb (27), suggesting that the natural class II molecular processing and antigen presentation pathways can be simulated in these cells (**Figure 1D**). As for human class II molecules, co-expression of human CD74 results in much higher surface expression of human DQ molecules (**Figure 1E**). We conclude that K528 is a versatile parental cell line for surface expression of MHC class I and II molecules from different mammalian species.

### Multiplex Detection of Anti-HLA Antibodies in Serum Samples From Allo-Sensitized Patients

**Figure 2:**
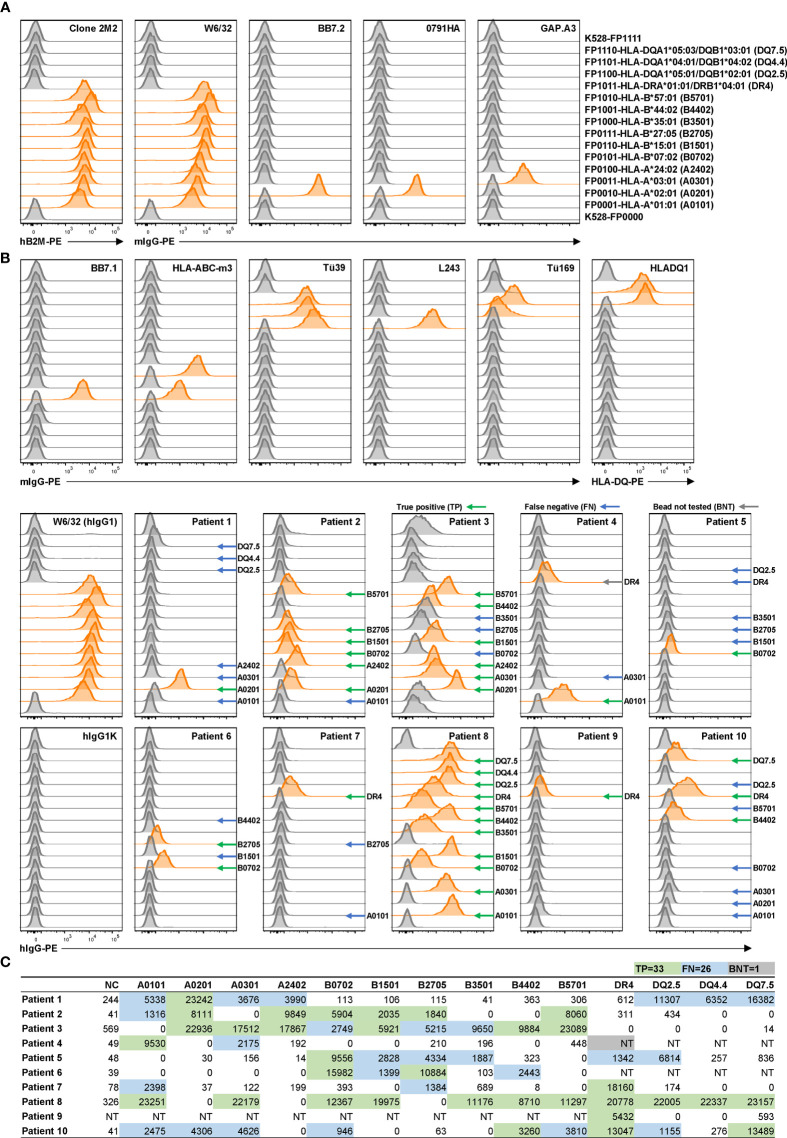
Application of reporter cell line assay in multiplex detection of anti-HLA antibodies in serum samples from allo-sensitized patients. **(A)** K528-derived fluorescence-barcoded reporter cell lines were transduced to express HLA class I molecules along with human β2M or class II molecules along with human CD74. **(A)** The resultant cell lines were pooled, and the surface expression of HLA molecules on individual cell lines were validated with a panel of reference monoclonal antibodies. Example gating strategy is shown in **Figure S3**. Demultiplexed reporter cell lines with MFI values above three-fold of the average MFI value of internal control cell lines (K528-FP0000 and K528-FP1111) were scored as positive and the histograms were highlighted in orange. **(B)** Multiplex detection of anti-HLA antibodies with 10 serum samples from allo-sensitized patients. W6/32 (hIgG1) was used as positive control, and hIgG1κ and hIgG1λ (**Figure S4**) as negative controls in staining. Positive readouts that are also positive in clinical HLA SAB readouts were considered as true positive (TP) and indicated with green arrows. Negative readouts that are positive in bead readouts were considered as false negative (FN) and indicated with blue arrows. Positive readouts that were bead not tested (BNT) in SAB assay are indicated with gray arrows. MFI values are listed in **Table S1**. **(C)** Clinical HLA SAB MFI readouts. The color codes are in accordance with those in B), with TPs in both assays highlighted in green, and TPs in SAB assay while FNs in reporter cell assay in blue. HLA alleles are listed in abbreviations as indicated in parentheses in **(A)**. For class II molecule lines, bulk cell line after antibiotic selection were used.

Using the newly generated K528 cell line, a basic panel of 16 fluorescence-barcoded sub-lines were generated in the same way as for K530 cell line (submitted). Briefly, K528 cells were transduced to express different combinations of four FPs which can be discriminated from each other by flow cytometry, resulting in 16 sub-lines with unique fluorescence barcodes. To establish a general tool for anti-HLA antibody screening, we designed a basic panel consisting of ten HLA class I and four class II molecules which are highly prevalent in our patient population. Class I genes were transduced into barcoded cell lines along with human β2M whereas class II genes were incorporated along with human CD74. The resultant HLA-expressing reporter-cell panel was subsequently validated for specificity using a panel of HLA-specific monoclonal antibodies. All 14 HLA-expression cell lines were pooled along with two internal control cell lines without HLA transgene but express all or none of the barcoding FPs, respectively. The pooled reporter cells were then labeled with HLA-specific reference antibodies individually followed by a PE-conjugated secondary antibody. The labeled and washed cells were then examined for FP and PE signals by flow cytometry. Individual reporter cell lines were demultiplexed by gating for specific combinations of FP expression (**Figure S3**). Binding activity by each HLA reference antibody is proportional to the median fluorescence intensity (MFI) for each reporter cell line in the PE detection channel. In consequence, the specificity of each reference HLA antibody can be determined within the reporter cell line panel used for screening. In all cases, the binding patterns of each reference antibody matched its published specificity (**Figure 2A**).

This reporter cell panel permitted detection of anti-HLA antibodies present in serum samples from allo-sensitized patients. Ten blinded, serum samples were tested (**Figure 2B**, **Figure S4**) and the FACS MFI readouts compared to corresponding Luminex® readouts from a clinical HLA SAB assay (**Figure 2C**). No HLA antibodies detected by the reporter cell assay were absent in the clinical SAB assay, confirming the high specificity of the reporter cell assay. In contrast, for 59 true positive (TP) readouts in the SAB assay, only 33 were detected in the reporter cell assay. Most (22/26, 85%) of the discrepancies correlated with lower bead MFI values when compared to HLA alleles that tested positive with the reporter cell assay. These discrepant results may reflect lower sensitivity of the reporter cell assay due to the lower density of HLA antigen on the cell surface compared to beads. This lower density would result in lower signals, especially when the relevant antibody is at low concentration in the serum (28). It is also possible that these “false negatives” in the reporter cell assay reflect spurious antibody binding in the SAB assay due to the presence of denatured HLA antigens (29–31). However, for this study and in clinical practice, this type of spurious background binding is mitigated by the use HLA pattern analysis to determine TP SAB reactions. We conclude that the multiplex reporter cell assay likely exhibits lower sensitivity than the clinical SAB assay. If this is the case, its application should be limited to conditions when the concentration of relevant antibody is not limiting, *e.g.*, with monoclonal antibodies or single B-cell culture supernatants. In the case of single B-cell cultures, the clonal antibodies in most (≥ 90%) culture supernatants reach concentrations above 1 µg/ml (11). Under these conditions, the reporter cell assay is an economic and effective alternative to clinical SAB assay in high-throughput screening.

### Multiplex Detection of Mamu Antibodies in Serum Samples From Sensitized Rhesus Macaques

**Figure 3:**
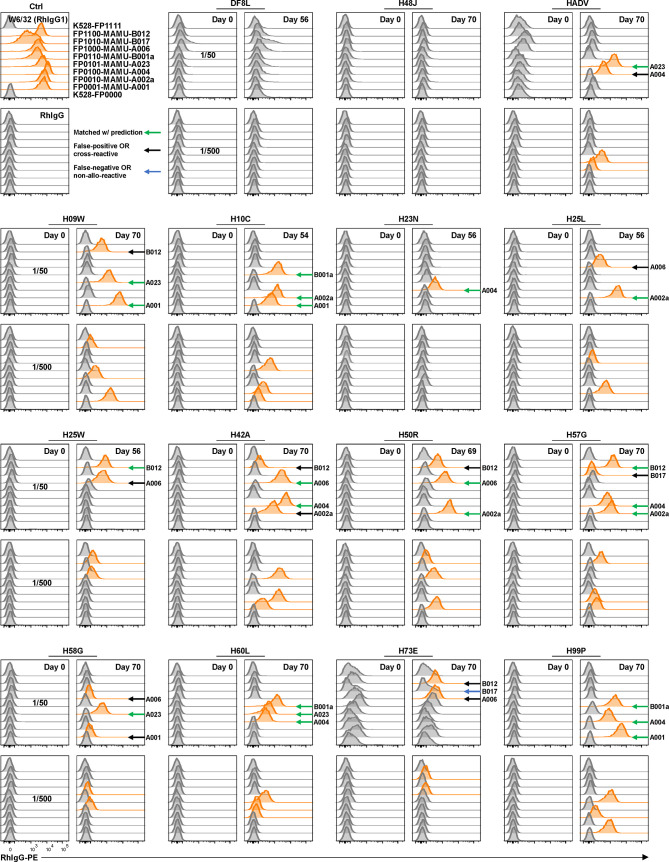
Application of reporter cell line assay in multiplex detection of anti-Mamu antibodies in serum samples from allo-sensitized RMs. K528-derived fluorescence-barcoded reporter cell lines were transduced to express Mamu class I molecules along with rhesus β2M. The resultant monoclonal cell lines were pooled, stained with sera from 15 allo-sensitized RMs at dilutions 1/50 and 1/500. W6/32 (RhIgG1) was used as positive control, and normal rhesus serum IgG (RhIgG) as negative control in staining. Demultiplexed reporter cell lines with MFI values above three-fold of the average MFI value of internal control cell lines (K528-FP0000 and K528-FP1111) were scored as positive and the histograms were highlighted in orange. The specificities were predicted based on donor Mamu haplotypes that mismatch with host ones. Positive readouts matched with prediction were indicated with green arrows. Positive readouts beyond prediction were either false positive detections or due to cross-reactivity and were indicated with black arrows. Predicted but undetected specificities were either false negative detections or due to non-allo-reactivity of the donor Mamu molecules in the corresponding host, and were indicated with blue arrows. B012 stands for B012a or B012b.

Rhesus macaques (RMs) are a valuable animal model in many pre-clinical studies, including transplantation. However, specific reagents for studies with RMs are limited. To our knowledge, only one allele-specific Mamu antibody has been reported (32) and there is no single Mamu antigen bead panel available commercially. The multiplex reporter cell platform we established for HLA is readily adaptable to Mamu antigen expression. To demonstrate this potential, a panel of class I Mamu molecules and rhesus β2M were expressed in FP-barcoded reporter cell lines by transduction. Robust surface expression of Mamu proteins was confirmed using the cross-reactive antibody, W6/32 (**Figure 3**). To validate the specificity and utility of this reporter cell panel, we tested 15 blinded serum samples from allo-sensitized RMs following two sequential skin transplants (12) for the presence of allo-Mamu antibodies. We predicted the elicited antibody reactivities based on donor Mamu haplotypes that were mismatched with recipient haplotypes, and compared these to the binding activities observed in the reporter cell assay. As shown in **Figure 3** and **Table 1**, except for a single case in RM H73E, we detected binding activities for all the predicted antibody responses (23/24, 96%). This single negative result is either a false negative due, perhaps to the relative low density of Mamu proteins expressed by the reporter cells, or to the non-responsiveness of the particular allo-antigen in this host RM.

**Table 1:** Mamu haplotypes of recipient and donor RMs and reporter cell line detection results.

RM ID	Recipient haplotype	Donor haplotype	Matched with prediction	False positive OR cross-reactive	False negative OR non-allo-reactive
DF8L	A004/A025/B012b/B017a	A007/A701b/B068/B085	—	—	—
H48J	A004/A023/B002/B043a	A019/A019/B015c/B015c	—	—	—
HADV	A025/A002a/B012b/B001a	A019/**A023**/B015c/B043a	A023	A004	—
H09W	A006/A004/B024a/B015a	**A001**/**A023**/B055/B043a	A001, A023	B012	—
H10C	A004/A025/B012b/B048	**A002a**/**A001**/B012a/**B001a**	A001, A002a, B001a	—	—
H23N	A016/A025/B001a/B012b	**A004**/A019/B056b/B015c	A004	—	—
H25L	A004/A008/B012b/B069b	A018a/**A002a**/B002/B055	A002a	A006	—
H25W	A004/A002a/B028/B055	A008/A008/B048/**B012b**	B012	A006	—
H42A	A001/A023/B055/B043a	**A006**/**A004**/B024a/B015a	A004, A006	A002a, B012	—
H50R	A004/A001/B048/B047a	**A002a**/**A006**/B015a/B055	A002a, A006	B012	—
H57G	A023/A074/B043a/B001a	**A004**/**A002a**/B001a/**B012a**	A002a, A004, B012	A006	—
H58G	A004/A002a/B001a/B012a	**A023**/A074/B043a/B001a	A023	A001, A006	—
H60L	A002a/A105/B012a/B002	**A004**/**A023**/**B001a**/B043a	A004, A023, B001a	—	—
H73E	A001/A004/B001a/B069a	A008/A025/B015b/**B017a**	—	A006, B012	B017
H99P	A002a/A002a/B015a/B012a	**A001**/**A004**/**B001a**/B012b	A001, A004, B001a	—	—

Donor haplotypes mismatching with recipient haplotypes, which had a matched reporter cell lines in the detection panel and would therefore be considered as a predicted positive readout, are highlighted in bold. B012 stands for B012a or B012b.

On the other hand, 12 binding signals beyond the predicted reactivities were detected. These positive readouts could be false positive results due to expression artifacts in the reporter cells, or – and we believe more likely - the result of cross-reactive epitopes shared between Mamu allo-antigens. Unfortunately, validated reagents to rule out either of these two possibilities do not exist; however, in view of the high specificity of the HLA reporter cell assay above (**Figures 2A, B**), and considering the well documented cross-reactive epitopes shared between HLA molecules (**Figure 2C**), we believe that these positive readouts represent true cross-reactivities in allo-sensitized RMs. The potential identification of novel, cross-reactive Mamu epitopes, highlights the utility of this multiplex reporter cell assay in experimental models where multiplex bead reagents are unavailable or difficult to generate.

Of note, serum samples from RM HADV at Day 0 and from H73E at Days 0 and 70 exhibited high background staining of all reporter cell lines including the internal control lines bearing no MHC molecules; with dilution this polyreactivity became negligible (compare serum dilutions of 1/50 to 1/500). Our records show no significant differences in pre-sampling treatments for these animals and we were able to exclude technical issues in the binding assay. Consequently, we consider this serum polyreactivity to reflect intrinsic properties of the animals or samples. Possible explanations include 1) presence of polyreactive, presumably low affinity, serum IgG, 2) serum IgG antibody specific for an unknown antigen(s) present on the parental K528 cells, and 3) serum components, e.g., complement-decorated antibody/antigen complexes, which mediate non-specific binding of antibodies to the reporter cells.

### Evidence for Affinity Maturation in an HLA-Specific Bmem Cell From a Sensitized Patient

We established an antigen-specific single-cell culture system recently (11, 20, 33, 34), which supports robust proliferation of single Bmem cells (to ≈90,000 daughter cells by day 25) followed by plasmacytic differentiation and IgG production, yielding an average 50 µg/ml of clonal antibody in culture supernatants. This robust production of clonal IgG enables high-throughput screening for MHC-specific clones using the reporter cell assay described in this report. With this culture system, the V(D)J rearrangements encoding MHC-specific BCRs can be readily recovered by PCR amplification, sequenced, and expressed as recombinant antibodies (rAbs) for further characterization.

**Figure 4:**
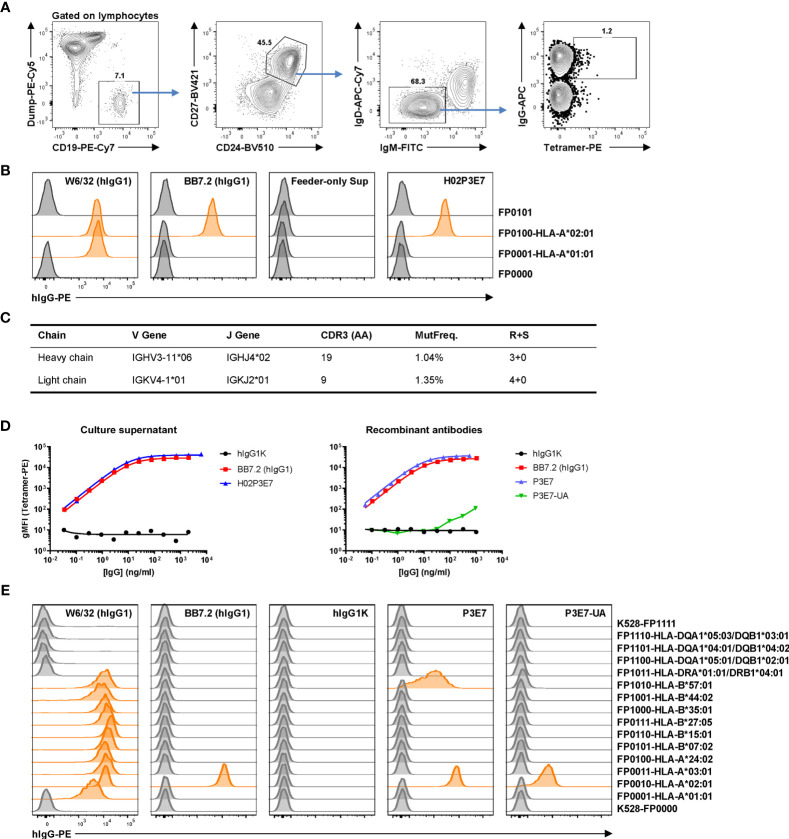
Identification and characterization of an anti-allo HLA Bmem cell clone from a sensitized patient. **(A)** Gating strategy for antigen-specific single B-cell sorting. Frozen-thawed PBMCs from an allo-sensitized patient was stained with HLA-A*02:01 tetramer and surface markers to identify tetramer-binding IgG^+^ Bmem cells for single B-cell culture. Dump, CD3/CD14/CD16/7-AAD. After 25 days culture, IgG^+^ supernatants were subjected to a reporter cell assay to screen for HLA-binding clones. **(B)** Example data of reporter cell assay with positive control antibodies, a negative control (feeder-only) supernatant and supernatant from the single HLA-A*02:01-binding clone H02P3E7 identified. In this experiment, two HLA-expressing and two control reporter cell lines were used. W6/32, pan class I HLA specific; BB7.2, HLA-A*02-specific. **(C)** Genetics of the BCR of the identified clone H02P3E7. MutFreq, nucleotide mutation frequency of V genes. R+S, replacement and silent mutations. **(D)** Binding activities of the culture supernatant (left panel) from Bmem clone H02P3E7 and rAbs (right panel) from identified clone (P3E7) and inferred germline clone (P3E7-UA) to HLA-A*02:01 tetramer antigen. hIgG1K, human IgG1 kappa isotype control antibody. **(E)** Reporter cell assay with control antibodies and rAbs P3E7 and P3E7-UA. Demultiplexed reporter cell lines with MFI values above three-fold of the average MFI value of internal control cell lines (K528-FP0000 and K528-FP1111) were scored as positive and the histograms were highlighted in orange.

We employed this single-cell culture method to isolate and characterize HLA-specific Bmem cells and to generate HLA rAbs from an allo-sensitized patient. The patient was sensitized by three pregnancies and a history of transfusion. Serological HLA-A*02:01 reactivity was detected by SAB as the immunodominant or strongest antibody as measured by mean fluorescent intensity (MFI). PBMCs from this patient were labeled with A*02:01 tetramer along with surface markers (CD19^+^ CD27^+^ CD24^hi^ IgD^–^ IgM^–^ IgG^+^) to identify Bmem cells. Tetramer^+^ IgG^+^ Bmem cells (**Figure 4A**) were sorted into single B-cell cultures as described (11). After 25 days of culture, a cloning efficiency of 40% (270/672) was determined by screening for secreted IgG in culture supernatants. IgG^+^ supernatants were then tested for HLA binding activity using the HLA reporter cell assay. One clone, H02P3E7, exhibited avid binding to A*02:01 but not to A*01:01 (**Figure 4B**).

We sequenced the V(D)J rearrangements present in this HLA-A*02:01 specific clone by amplifying the human IgG heavy- and light-chain genes from the expanded, clonal B-cell population in the corresponding culture (15, 35). The recovered and sequenced BCR genes carried mutations (>1%) in both the VH and VL gene segments (**Figure 4C**), consistent with this IgG^+^ Bmem cell clone being derived from a germinal center (GC) response. A recombinant antibody (rAb), P3E7, was generated from the V(D)J sequences present in the cloned Bmem cell H02P3E7 and a second, P3E7-UA, from the inferred, germline V(D)J sequences expressed by the unmutated ancestor [UA, (36)] of the H02P3E7 Bmem cell. The binding of both rAbs to A*02:01 was confirmed in a Luminex® assay using A*02:01 tetramer as the antigen target (**Figure 4D**). The binding avidity of rAb P3E7 was comparable to that of BB7.2, a hybridoma derived A*02:01-specific antibody, and has a calculated Kd value of 68.5 pM. Interestingly, the binding activity of the rAb P3E7-UA to A*02:01 was decreased by 4-logs compared to the mutated rAb P3E7. In a reporter cell assay (**Figure 4E**), we confirmed that both rAbs can bind to cell-surface expressed A*02:01 antigens, with P3E7 showing much more avid binding activity than P3E7-UA does. Moreover, P3E7 also shows binding activity towards B*57:01, suggesting that this antibody binds to a shared epitope in these two alleles. This observation supports the notion that in the sensitized patient, this Bmem cell resulted from HLA-specific affinity maturation in a GC prior to its entry into the Bmem compartment.

**Figure 5:**
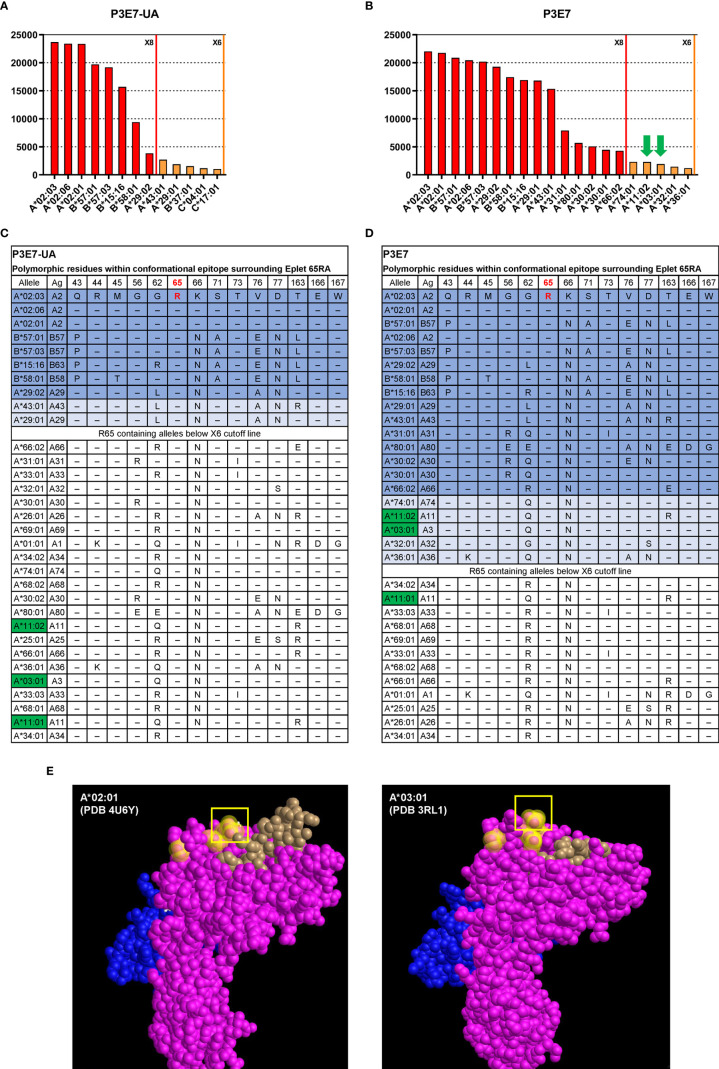
Interrogation of HLA-A*02 specific recombinant antibodies isolated from an allo-sensitized patient. HLA binding specificities of rAbs P3E7-UA **(A)** and P3E7 **(B)** using a Luminex^®^ single HLA antigen bead 100-plex panel. Alleles with MFI values above the X6 threshold (MFI > 1000) are shown. Autologous alleles are indicated with green arrows. **(C, D)** HLA alleles sharing eplet 65RA and the polymorphic amino acids within this potential 15 Å conformational epitope. Alleles are listed in the order of MFI values from high to low. Alleles above the X8 threshold (MFI > 3000) are shaded in dark blue and alleles above the X6 threshold in light blue. Autologous HLA alleles are highlighted in green. **(E)** Three-dimensional structure of allogeneic (A*02:01) and autologous HLA structures from published data with PDB ID indicated. HLA α chain (pink), β2M (blue) and bound peptide (brown) are visualized using Cn3d software. Residues 56, 62, and arginine at position 65 (box) are highlighted yellow.

In addition, we determined the HLA allele binding patterns of rAbs P3E7 and P3E7-UA using a clinically validated HLA class I 100-plex SAB panel. The threshold for positivity was determined by examining MFI values for the negative controls and the point at which the HLA allele binding pattern was interrupted by non-related HLA-B and -C alleles. Tests of the unmutated P3E7-UA rAb displayed strong reactivity for beads bearing HLA-A*02 and B*57 alleles (23,679 - 19,171 MFI), lower binding to B*15:16 (B63) and B*58:01 (15,685 - 9,377 MFI), and the lowest binding to A*43:01 and A*29 alleles (3,815-1,866 MFI) (**Figure 5A**). Using the HLA Epitope Registry (https://www.epregistry.com.br/), we identified a common HLA amino-acid motif or “eplet” shared across the HLA alleles to which the rAbs bound. The 10 bound HLA alleles share homology within a potential 15 angstrom conformational epitope surrounding an arginine residue at amino acid position 65 defined by HLA eplet 65RA (**Figures 5B, E**). As expected, stronger reactivity was observed for the mutated P3E7 rAb compared to the unmutated P3E7-UA (**Figures 5A, D**) along with an extended binding pattern to include 13 additional HLA alleles also containing HLA eplet 65RA.

Bordering amino acid differences influence antibody binding to HLA conformational epitopes; binding variability, however, may also reflect the quality and density of HLA molecules on individual Luminex® beads. Simultaneous comparison of P3E7 and P3E7-UA binding at identical concentrations allowed us to control for bead differences and examine how BCR mutation affected BCR affinity and specificity. Our analysis revealed that affinity maturation of P3E7 increased binding to non-HLA-A*02 alleles with variable residues at position 45 and 62 increasing MFI values (>15,000) for HLA-B*58, A*29, B*15:16 and A*43 from that observed for the progenitor P3E7-UA antibody (**Figure 5B**). This expanded reactivity also allowed binding to additional HLA alleles bearing the 65RA eplet but with substantial variability about this conformational epitope. Interestingly, in the course of its somatic evolution, the H02P3E7 Bmem cell acquired the capacity to recognize HLA alleles (A*11:01 and A*03:01) present within the patient; this auto-reactivity was not present in the germline P3E7-UA rAb. When P3E7 was tested at a higher concentration (6x) the same reactivity pattern was maintained and extended to include A*68, A*69, A*01, and A*33:01 and correspondingly higher (> 6,000) MFI values were observed for the autologous HLA alleles as well (data not shown). The location of eplet 65RA on allogenic HLA-A*02:01 [Protein Data Bank (PDB) ID 4U6Y (37)] and autologous A*03:01 [PDB ID 3RL1 (38)] structures are shown in (**Figure 5E**). Therefore, the process of affinity maturation in this patient was coupled to the generation of humoral autoreactivity.

## Discussion

In our report of new experimental tools for monitoring of MHC-sensitized recipients, we have demonstrated how FP barcoded cells expressing HLA or Mamu proteins can be used to detect and characterize specific antibody and B cells. Our data describe a method for isolation of individual MHC-specific Bmem cells and their characterization by panels of MHC reporter cells. Using this single cell method, we can interrogate HLA antibody-secreting clones for their BCR sequences, infer clonal relationships between mutated Bmem cells and UAs, and demonstrate affinity maturation as a process of clonal evolution. Lastly, by the generation of representative rAbs, we demonstrate the utility of SAB Luminex® panels and HLA eplet analysis to define HLA conformational epitopes recognized by individual B cells and the trajectory of their evolution by somatic mutation.

It is notable that this technology/method can benefit in nonhuman primate (NHP) research since currently there are no available means to evaluate individual Mamu (or Mafa) specific antibodies. Non-human primates offer an important experimental model to develop novel therapies for clinical translation (39). Attempts to use HLA-specific SAB reagents to characterize NHP alloantibody responses have been made (40), albeit with limited resolution of specificity. In most NHP research, therefore, the standard assay for alloantibody detection relies on a donor-cell antibody capture method analogous to the flow cytometric crossmatch used in clinical transplantation (12, 41, 42). Unfortunately, antigen-specific alloantibody quantitation, such as the HLA SAB Luminex® assay provides, has not been realized, despite conserved epitopes permitting detectable positivity (43). Our use of FP barcoded cells expressing Mamu antigens (**Table 1**) allows for multiplex detection of multiple alloantibody specificities within the same sample, with good detection of alloantibody directed against sensitizing antigens. The additional positivity detected in the Mamu reporter assay may reflect false positives but, as the single-cell human rAb data suggest, more likely represents unknown cross-reactive binding across similar epitopes. Since this degree of alloantibody specificity has not been widely available these new tools allow for a greater understanding of shared epitopes, binding patterns, hierarchy among Mamu (or Mafa) antigens.

In human studies, the utility of HLA expressing reporter cells may be limited to that of a screening tool, given the apparent greater sensitivity of the SAB Luminex®; assay. For research purposes, however, SAB testing with commercially available products can be prohibitively expensive. For example, in the case of single-cell cultures in which hundreds or thousands of individual culture wells contain clonal IgG, SAB bead analysis of each well could present a daunting expense. As for the analogous Luminex®; bead assay, the barcoding of each HLA or Mamu expressing cell line with a unique pattern of FPs permits multiplexing of the assay in a single tube, limiting the volume of sample necessary for antibody detection.

In conjunction with our barcoded reporter cell lines, we used a single human B-cell culture system (11, 20, 33, 34) to isolate and characterize HLA-specific Bmem cells from an allo-sensitized patient. In previous studies, we sorted hemagglutinin-binding Bmem cells from vaccinated subjects into single-cell cultures and about half of the cultured Bmem cells produced hemagglutinin-specific IgG (11). In this study, however, from 270 cultures of Bmem cells identified by HLA-A*02:01 tetramer binding only a single clonal culture produced HLA-specific IgG (**Figure 5B**). The low frequency of HLA-specific clones among tetramer-binding B cells is likely the consequence of the low specificity of the antigen-tetramer “hook” and perhaps, the absolute frequency of HLA-specific B cells. As HLA-specific Bmem cells are usually at low frequency in allo-sensitized patients (10, 44), development of high-quality HLA antigen hooks will be critical for efficient isolation of specific Bmem cells.

With the single HLA-specific B cell clone identified, we determined the BCR V(D)J sequences and identified mutation frequencies of about 1% in the V genes of both the heavy- and light chain gene rearrangements (**Figure 4C**). We confirmed the avid binding of the A*02:01 tetramer by secreted clonal IgG with a recombinant antibody prepared from the recovered BCR gene sequences. In addition, the inferred germline progenitor (UA) of the recovered Bmem clone was also generated and comparison of P3E7 and the UA recombinant antibodies provided evidence for an avidity increase of some 10^4^-fold as a consequence of somatic hypermutation (**Figure 4D**). These data, and the Bmem phenotype recovered by flow sorting (**Figure 4A**), suggest strongly that this B cell clone was a typical class-switched Bmem cell derived from a GC response after successful rounds of affinity maturation. This is an example, albeit a single one, of HLA-specific Bmem cells in sensitized patients. Moreover, the strong binding avidity of the mature rAb implies that the H02P3E7 Bmem cell would likely be capable of differentiating into a plasma cell (45), and to secrete HLA antibody on rechallenge. Antibody of this affinity would impose a threat of an acute AMR after transplantation even if HLA serum antibody titers were negligible prior to transplant (44). This observation highlights the significance of monitoring of HLA Bmem cells in pre-transplant patients in clinic.

An interesting observation with the HLA Bmem recovered is that its mutated and affinity-matured BCR acquired autoreactivity absent in its germline precursor. Whereas the inferred germline antibody was specific for the products of several allo-HLA-A*02 alleles, the more avid BCR expressed by the Bmem H02P3E7 daughter cell acquired the capacity to bind autologous HLA molecules in the course of its somatic evolution (**Figure 5**). The acquisition of self-reactivity is known to be associated with human Ig-class recombination (35) and, presumably, clonal maturation in GCs. Nonetheless, most current models for GC responses posit negative selection to limit the generation of autoimmunity by AID-mediated hypermutation (46–49). Indeed, we observed the opposite of what Goodnow and colleagues have named clonal redemption (47, 48). Rather than the recruitment of autoreactive B cells into GCs where they lose autoreactivity in the process of affinity maturation to an exogenous antigen, the naïve P3E7-UA cell was presumably recruited to a GC on exposure to allo-HLA and there its progeny acquired reactivity to self-HLA in conjunction with allo-specific affinity maturation (**Figure 5**). Surprisingly, this *de novo* autoreactivity was not against some rare or cryptic self-antigen but to universally expressed HLA class I determinants. While it is possible that the binding activities to self HLA molecules detected in the SAB assay are not physiologically relevant, it will be interesting to determine if the relevant membrane associated HLA molecules trigger BCR signaling in B cell lines harboring the matured P3E7 BCR. Additional study will be needed to determine whether autoreactivity is a common phenomenon among allo-HLA Bmem cells.

These new cell tools permit multiplex detection of allo-MHC antibody in humans and non-human primates. The utility of this approach is limited only by the number of FP barcoded cell lines and has the potential of multiplexing up to 256 distinct cells for antibody detection in single samples. Additionally, we have demonstrated the application of barcoded reporter cells in screening for HLA-specific antibody production by single-cell cultures of human Bmem cells. In this way, we characterized and expressed rAb derived from a human Bmem cell, demonstrating its somatic evolution by mutation and selection for increasing affinity. We rapidly identified the 65RA eplet as the putative epitope for the Bmem BCR using SAB HLA-Luminex® assays. These combinations of new and established techniques offer increased understanding of humoral responses to allo-MHC.

## Data Availability Statement

The datasets presented in this study can be found in online repositories. The names of the repository/repositories and accession number(s) can be found below: GenBank accessions MZ152901, MZ152902, MZ152903, MZ152904.

## Ethics Statement

The studies involving human participants were reviewed and approved by Duke Institutional Review Board Committee. The patients/participants provided their written informed consent to participate in this study. The animal study was reviewed and approved by Duke University Institutional Animal Care and Use Committee.

## Author Contributions

SS, SK, and GK contributed to conception and design of the study. SS, MM, JK, AJ performed the experiments described in this study. SK and GK helped to review and trouble-shoot all experiments. SS, MM, JK, and AJ wrote specific sections of the initial manuscript. All authors contributed to the article and approved the submitted version.

## Funding

The research was partially supported by National Institutes of Health grant R01 AI128832 (to GK), and by the National Institute of Allergy and Infectious Diseases of the National Institutes of Health as part of the NHP Transplantation Tolerance Cooperative Study Group under the U19AI131471 (to SK). The content is solely the responsibility of the authors and does not necessarily represent the official views of the National Institutes of Health. HLA-Reporter cell line development was supported by the Immune Tolerance Network and supported by the National Institute of Diabetes and Digestive and Kidney Diseases (NIDDK) and the National Institute of Allergy and Infectious Diseases (NIAID) of the National Institutes of Health under Award Number UM1AI109565. The content is solely the responsibility of the authors and does not necessarily represent the official views of the National Institutes of Health.

## Conflict of Interest

The authors declare that the research was conducted in the absence of any commercial or financial relationships that could be construed as a potential conflict of interest.
