# Supplementary material for: Allo-Specific Humoral Responses: New Methods for Screening Donor-Specific Antibody and Characterization of HLA-Specific Memory B Cells
Source: Front Immunol. 2021 Jul 13;12:705140. doi: 10.3389/fimmu.2021.705140 (PMC8313870; doi:10.3389/fimmu.2021.705140)
Supplement: Supplementary file 1 [file Table_1.docx]

Supplementary Material

**Figure S1.** **The generation of K528 cell line.** K530 cell line was engineered to knockout *B2M* gene by CRISPR-Cas9 mediated gene targeting. **(A)** The design of sgRNAs targeting coding region in exon 1 of human *B2M* gene (Genbank accession NG_012920). Exon 1 is indicated with boxes and the start codon is highlighted in yellow. sgRNA sequences are highlighted in cyan with corresponding PAM sequences in red. Primers hB2M-gF/hB2M-gR were used in amplification and hB2M-gF in sequencing. **(B)** FACS detection of intracellular β2M expression. sgRNA groups are bulk cell culture after Cas9-sgRNA vector transfection and puromycin selection. K528 is a monoclonal cell line derived from sgRNA-hB2M-1 transfected cells by single-cell flow cytometry sorting. **(C)** Sequence analysis of targeted region in the selected cell line K528. A single homogeneous modified sequence was identified from both bulk amplicon and cloned plasmid sequencing. Cyan box shows sgRNA region, red the PAM sequence. The blue arrow indicates a single inserted nucleotide.

**Figure S2. Morphological gating of cell lines for the analysis of expression of surface and intracellular markers.** Example date corresponding to Figure 1A are shown. The major population of live cells were gated, which accounts for >85% of total events acquired on the flow cytometer. The only exception is the sample of Raji cells stained with DR/DP/DQ-PE-Cy7 antibody (clone Tü39). The staining with this antibody, but not with the isotype control antibody, resulted in presumably cell activation and death. In this case, the gating was set up based on the same cell line stained with the isotype control antibody.

**0110 0111**

**0100 0101**

**0010 0011**

**0000 0001**

**1110 1111**

**1100 1101**

**1010 1011**

**1000 1001**

**10 11**

**00 01**

**Figure S3. Gating strategy for demultiplexing of pooled fluorescence barcoded reporter cell lines.** Total live cells were gated and plotted for fluorescent proteins (FPs) mNeonGreen (mNG) and mCardinal (mCar) expression, resulting in four distinct populations: mNG^–^mCar^–^, mNG^–^mCar^+^, mNG^+^mCar^–^ and mNG^+^mCar^+^. Binary barcode for each population is indicated in each quadrant. For each population, the expression of EBFP2 and mTurquoise2 (mTq2) were further plotted, yielding four well-separated sub-populations: EBFP2^–^mTq2^–^, EBFP2^+^mTq2^–^, EBFP2^–^mTq2^+^ and EBFP2^+^mTq2^+^. Binary barcode for each sub-population is indicated in each quadrant. These 16 sub-populations represent the 16 individual reporter cell lines before pooling. Antibody binding to each subpopulation can then be analyzed by gating on each of them. Example data from the sample stained with antibody W6/32 in Figure 2A are shown.


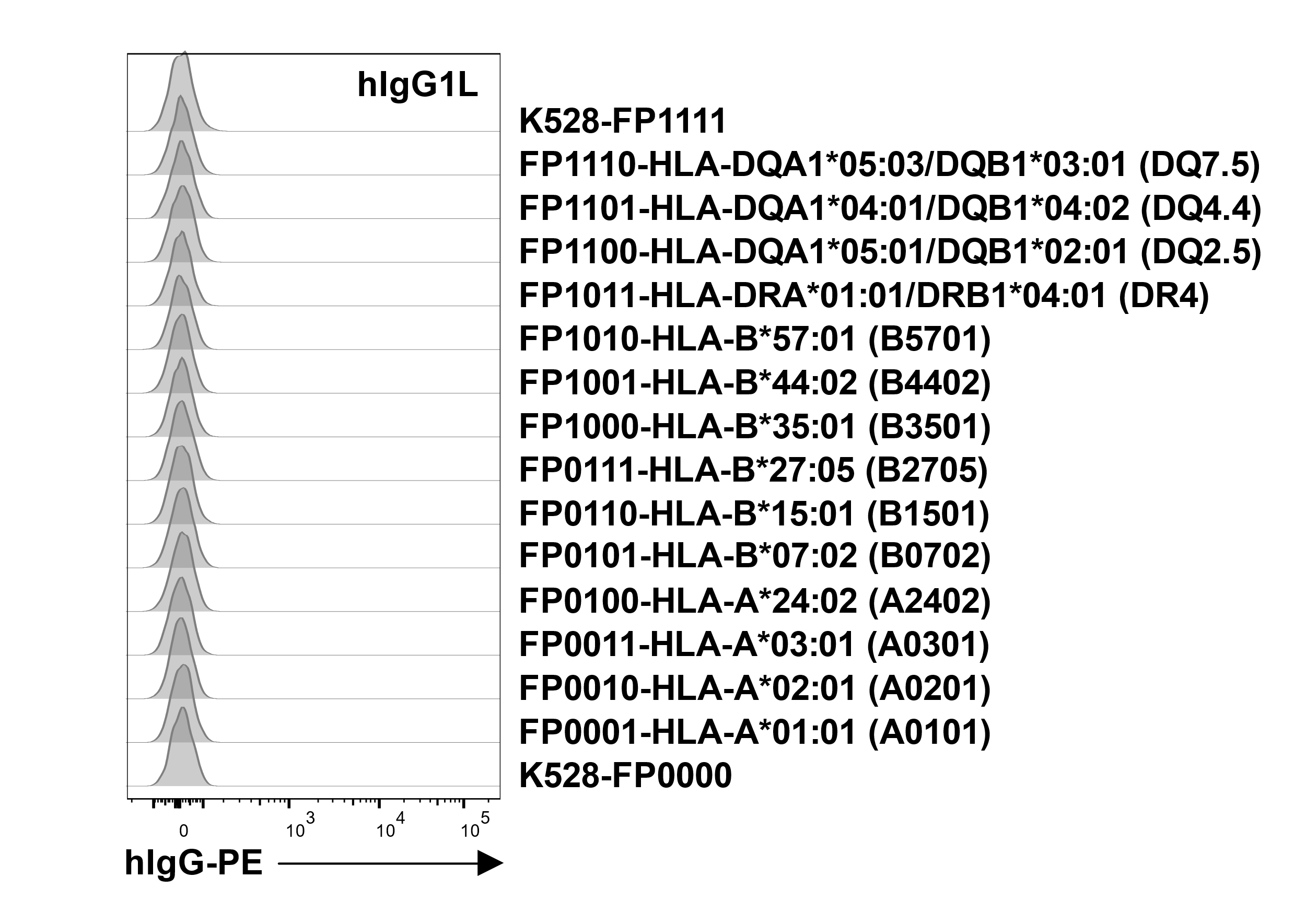


**Figure S4. Histograms showing the staining of HLA-expressing reporter cell panel with human IgG1λ isotype control antibody.** Derived from the same experiment and analyzed in the same way as shown in Figure 2B.

**Table S1. MFI values of HLA-expression reporter cell lines stained with sensitized patient serum samples (Figure 2B)**

|  | **FP0000** | **FP1111** | **Cutoff** | **A0101** | **A0201** | **A0301** | **A2402** | **B0702** | **B1501** | **B2705** | **B3501** | **B4402** | **B5701** | **DR4** | **DQ2.5** | **DQ4.4** | **DQ7.5** |
| --- | --- | --- | --- | --- | --- | --- | --- | --- | --- | --- | --- | --- | --- | --- | --- | --- | --- |
| **Patient 1** | 35 | 36 | 106 | 98 | 985 | 55 | 52 | 32 | 36 | 42 | 43 | 44 | 44 | 28 | 45 | 48 | 100 |
| **Patient 2** | 52 | 96 | 155 | 65 | 239 | 74 | 371 | 158 | 204 | 170 | 52 | 61 | 190 | 53 | 48 | 45 | 60 |
| **Patient 3** | 170 | 253 | 509 | 235 | 6176 | 906 | 749 | 157 | 812 | 282 | 389 | 562 | 2553 | 155 | 144 | 110 | 137 |
| **Patient 4** | 57 | 68 | 187 | 695 | 60 | 97 | 72 | 77 | 63 | 66 | 73 | 82 | 92 | 191 | 64 | 54 | 59 |
| **Patient 5** | 37 | 27 | 96 | 32 | 25 | 24 | 28 | 118 | 75 | 71 | 57 | 34 | 33 | 25 | 44 | 32 | 30 |
| **Patient 6** | 25 | 21 | 68 | 21 | 17 | 17 | 24 | 245 | 35 | 125 | 20 | 18 | 22 | 18 | 21 | 26 | 22 |
| **Patient 7** | 44 | 50 | 141 | 45 | 48 | 43 | 50 | 44 | 43 | 62 | 56 | 78 | 46 | 227 | 42 | 45 | 48 |
| **Patient 8** | 32 | 79 | 165 | 3963 | 24 | 2155 | 32 | 278 | 3400 | 35 | 339 | 2048 | 284 | 703 | 2650 | 2797 | 2810 |
| **Patient 9** | 30 | 26 | 83 | 80 | 30 | 31 | 36 | 24 | 33 | 29 | 30 | 31 | 31 | 99 | 43 | 36 | 50 |
| **Patient 10** | 41 | 54 | 142 | 57 | 85 | 102 | 43 | 43 | 33 | 42 | 38 | 195 | 94 | 481 | 37 | 37 | 211 |

The cutoff MFI values were determined as three-fold of the average MFI value of internal control cell lines (K528-FP0000 and K528-FP1111). MFI values shaded in grey were excluded from cutoff value calculation due to obvious higher background staining for this internal control than for the other internal control cell line and for other negatively stained HLA-expressing reporter cell lines (Figure 2B). Demultiplexed reporter cell lines with MFI values above the cutoff were scored as positive and highlighted in orange. HLA alleles were abbreviated as shown in Figure 2A.
